# Pulmonary arterial hypertension incidence in Latvia in 2019

**DOI:** 10.1002/pul2.12161

**Published:** 2022-10-01

**Authors:** Ricards Kaulins, Ainars Rudzitis, Aivars Lejnieks, Matiss Sablinskis, Andris Skride

**Affiliations:** ^1^ Department of Internal Diseases Riga Stradiņš University Riga Latvia; ^2^ Department of Rare Diseases Pauls Stradiņš Clinical University Hospital Riga Latvia; ^3^ Department of Internal Diseases Riga East Clinical University Hospital Riga Latvia

## INTRODUCTION

Pulmonary hypertension (PH) is a progressive pathophysiological disorder of abnormal elevation in pulmonary arterial blood pressure associated with poor prognosis.^1^ The clinical classification of PH is as follows: group 1—pulmonary arterial hypertension (PAH), group 2—PH due to left heart disease, group 3—PH due to lung disease and/or hypoxia, group 4—chronic thromboembolic PH, group 5—PH with unclear multifactorial mechanisms.^2^

PAH is a severe chronic cardiovascular condition caused by cellular proliferation and fibrosis of the small pulmonary arteries, resulting in a progressive increase in pulmonary vascular resistance (PVR).^3^, ^4^ PAH is considered a rare disease with reported incidences of 7.1−15 cases per million inhabitants (MI).^5^, ^6^, ^7^ In agreement with the current PAH clinical classification, PAH can be classified as idiopathic, heritable, induced by drugs or toxins, or associated with conditions such as connective tissue disease (CTD), congenital heart disease (CHD), portal hypertension, HIV infection, or schistosomiasis.^8^, ^9^ A definitive diagnosis of PAH requires hemodynamic demonstration at right heart catheterization of a mean pulmonary artery pressure (mPAP)  ≥ 20 mmHg, pulmonary artery wedge pressurE (PAWP)  ≤ 15 mmHg, and PVR > 3 Wood units.^10^

Since the creation of the National Institutes of Health registry for patients with “primary” PH in 1981, crucial advances have been made in epidemiology of the different subgroups of PH.^11^, ^12^, ^13^ Despite the fact that extensive national and international registries have generated important information on the epidemiology as well as have improved diagnosis and management of PAH patients, regional discrepancies are undoubtedly present in patient characteristics, diagnostic workup, and treatment availability. Various studies have shown that despite the recent advancements in the field of PAH, belated diagnosis of PAH is still a significant issue.^14^, ^15^, ^16^ This highlights the necessity for more epidemiological data from nationwide registries, which take into account local specificities to achieve a more detailed global perspective of PAH. Thus, annual reports on the characteristics of PAH patients can demonstrate the dynamics of PAH patients year over year and try to help to identify recent trends and patterns.

In this report, we present the incidence of PAH in Latvian adults in 2019 and their baseline characteristics including hemodynamics and exercise capacity.

## METHODS

This is a prospective, observational, study of the Latvian nationwide PH registry. All consecutive adult patients (≥18 years of age) recently diagnosed with PAH in the time period from January 1 to December 31, 2019 were included in the registry. A written informed consent for inclusion in the registry and publication of the study data was obtained from all patients after the nature of the study had been fully explained. This research was carried out in accordance with the 1975 Helsinki declaration (as revised in 2008). The diagnostic criteria for PAH patients were applied in accordance with latest 6th World Symposium on PH Task Force.^3^ To confirm the diagnosis of PAH, right heart catheterization was performed, measuring right atrial pressure (RAP), mPAP, PAWP, PVR, cardiac output (CO) and cardiac index (CI). All identified PAH cases were categorized into PAH subtypes according to the clinical classification as follows: idiopathic, familial, or associated with anorexigen exposure, CTD, CHD, portal hypertension, or HIV infection.^8^, ^9^

The initial risk stratification of enrolled PAH patients was performed analyzing noninvasive parameters World Health Organization PAH classification, demographics, estimated glomerular filtration rate, New York Heart Association (NYHA) Functional class, heart rate, all‐cause hospitalizations, systolic blood pressure, DLCO, 6‐min walking distance (6MWD), B‐type Natriuretic Peptide (BNP), echocardiography and two variables derived from the right heart catheterization (mean RAP [mRAP] and PVR). Patients were categorized as low, intermediate, or high risk, according to the method used by the Registry to Evaluate Early and Long‐Term PAH Disease Management Registry (REVEAL) Risk Score 2.0.^17^

Also, patient risk stratification was evaluated according to the european society of cardiology (ESC) simplified four‐strata risk‐assessment tool, evaluating World Health Organization Functional Classification (WHO‐FC), 6MWD and BNP, categorizing PAH patients into low, intermediate‐low, intermediate‐high and high risk groups, according to the recent guidelines.^2^

The obtained quantitative patient variables were described using mean, standard deviation (SD), minimum, and maximum.

## RESULTS

A total of 23 PAH patients were included in the registry, where 6 patients were male and 17 were female. The female/male ratio was 2.83. In accordance with the World Health Organization PAH classification, out of 23 diagnosed PAH patients, 15 (65.21%) patients were idiopathic PAH, 4 (17.39%) patients with PAH associated with CHD, 2 (8.70%) patients—PAH associated with CTD, 1 (4.35%) patient—PAH associated with portal hypertension and 1 (4.35%) patient—PAH associated with HIV infection. Table 1 shows the baseline clinical and hemodynamic characteristics as well as the comorbidities for Latvian PAH patients in 2019.

**Table 1:** Baseline clinical and hemodynamic characteristics of Latvian PAH patients in 2019

Parameter			
Number of patients		23	
Female/male ratio		2.83	

Baseline characteristics	**All patients (*n*** = **23), Mean** ± **SD**	**Male (*n*** = **6), Mean** ± **SD**	**Female (*n*** = **17), Mean** ± **SD**
Age, years	67.83 ± 11.99	62.67 ± 11.98	69.65 ± 11.81
BMI, kg/m^2^	31.83 ± 7.86	29.66 ± 6.03	32.47 ± 8.37
BNP, pg/ml	433.01 ± 476.68	526.10 ± 716.51	395.78 ± 389.48
SpO_2_, %	94.43 ± 4.93	96.00 ± 2.65	93.25 ± 6.29
Functional status			
NYHA class I/II/III/IV,%	4.35/8.70/65.22/21.73	16.67/0/66.66/16.67	0/11.76/64.70/23.54
6MWD, m	243.07 ± 126.77	280.00 ± 153.41	228.30 ± 120.50
Right heart catheterization			
mPAP, mmHg	43.73 ± 11.66	47.67 ± 11.66	42.25 ± 11.68
mRAP, mmHg	8.68 ± 7.68	11.67 ± 11.62	7.56 ± 5.72
PVR, Wood units	7.68 ± 4.04	7.39 ± 2.98	7.77 ± 4.40
PCWP, mmHg	10.64 ± 5.16	11.40 ± 2.07	10.39 ± 5.89
CI, l/min/m2 ± SD	2.44 ± 0.81	2.22 ± 0.81	2.50 ± 0.84
CO, l/min ± SD	4.51 ± 1.27	4.82 ± 1.60	4.43 ± 1.22

Abbreviations: BMI, body mass index; BNP, B‐type Natriuretic Peptide; CI, cardiac index; CO, cardiac output; mPAP, mean pulmonary artery pressure; mRAP, mean right atrial pressure; *n*, number; NYHA, New York Heart Association; PCWP, pulmonary capillary wedge pressure; PVR, pulmonary vascular resistance; SD, standard deviation; SpO_2_, oxygen saturation; 6MWD, 6‐min walking distance.

The mean age (±SD) of enrollment for PAH patients was 67.83 (±11.99) years, where the mean age for females was 69.65 (±11.81) and 62.67 (±11.98) for males. The mean oxygen saturation (SpO_2_) (±SD) at normal room air was 94.43 (±4.93), where males had a higher mean SpO_2_ 96.00 (±2.65) than females 93.25 (±6.29) at normal room air. Patients showed a mean (±SD) body mass index (BMI) of 31.83 (±7.86). According to the World Health Organization, BMI greater than or equal to 25 is defined as overweight and BMI greater than or equal to 30 as obese,^18^ thus 86.61% of PAH patients were overweight and 47.83% were obese at the time of enrollment. At the time of diagnosis, the 6MWD and the NYHA classification were accessed to describe exercise and functional capacity of the PAH patients. NYHA functional class I was present in 1 patient (4.35%), class II in 2 patients (8.70%), class III in 15 patients (65.22%) and class IV in 5 patients (21.73%). Meanwhile the mean (±SD) 6MWD for enrolled PAH patients was 243.07 (±126.77) meters, where the mean 6MWD for males was 280.00 (±153.41) and 228.30 (±120.50) for females. The BNP level in PAH patients was quite variable with a minimum of 32 pg/ml and maximum of 1592 pg/ml, showing a mean value (±SD) 433.01 (±476.68) pg/ml.

At the time of enrollment 13.0% of the patients were in the low‐risk group, 43.5% in the intermediate risk and 43.5% in the high‐risk group according to REVEAL risk score 2.0. Meanwhile according to the ESC simplified four‐strata risk‐assessment tool, patients were categorized as follows: intermediate‐low risk (*n* = 4, 17.4%), intermediate‐high risk (*n* = 16, 69.6%) and high risk (*n* = 3, 13.0%). The subsequent risk determinants in low, intermediate and high‐risk range and their proportions are presented in Table 2.

**Table 2:** Pulmonary Arterial Hypertension mortality risk of enrolled patients and their subsequent risk determinants in low, intermediate‐low, intermediate‐high and high risk range

	Low risk, *n* (%)	Intermediate‐low risk, *n* (%)	Intermediate‐high risk, *n* (%)	High risk, *n* (%)
Patients	0 (0)	4 (17.4)	16 (69.6)	3 (13.0)
Risk determinants				
WHO‐FC	3 (13.0)	0 (0)	13 (56.5)	7 (30.5)
6MWD	0 (0)	6 (26.1)	3 (13.0)	14 (60.9)
BNP	1 (4.4)	15 (65.2)	5 (21.7)	2 (8.7)

Abbreviations: BNP, B‐type natriuretic peptide; WHO FC, World Health Organization Functional Class; 6MWD, six min walking distance.

All of the 23 enrolled patients received PAH‐specific monotherapy of phosphodiesterase type 5 inhibitors (Sildenafil). Regarding comorbidities, systemic arterial hypertension was the most prevalent (73.9%) underlying condition, which was followed by atrial fibrillation (56.5%). PAH patient comorbidities and received treatment at the time of the enrollment among study patients are shown in Table 3.

**Table 3:** Concomitant diseases and received therapy at enrollment

Concomitant diseases	*n*, (%)
Systemic arterial hypertension	17 (73.9)
Atrial fibrillation	13 (56.5)
Dyslipidemia	7 (30.4)
Chronic kidney disease	3 (13.0)
Diabetes	3 (13.0)
Coronary heart disease	3 (13.0)
Thyroid disease	2 (8.7)
Obstructive sleep apnea	1 (4.4)
Chronic obstructive pulmonary disease	1 (4.4)
PAH‐specific treatment	
PDE5i	23 (100)
Treatment	
Beta blockers	20 (87.0)
ACEI	14 (60.9)
ARB	3 (13.0)
Potassium‐sparing diuretics	20 (87.0)
Loop diuretics	17 (73.9)
Thiazide diuretics	4 (17.4)
Acetylsalicylic acid	3 (13.0)
Statins	11 (47.8)
Anticoagulant	
Vitamin K antagonists	4 (17.4)
New oral anticoagulants	10 (43.5)

Abbreviations: ACEI, angiotensin convertase inhibitors; ARB, angiotensin receptor blockers; PDE5i, phosphodiesterase type 5 inhibitors.

In 2019, the number of inhabitants in Latvia was 1.91 million, where 1.56 million were ≥18 years old (http://data.csb.gov.lv, website accessed March 31, 2020). Thus, the estimated PAH incidence in Latvia in 2019 was 12.04 per MI and 14.74 per million adult inhabitants (MAI).

## DISCUSSION

The present registry represents the data form the Latvian nationwide PH registry obtained in 2019, which was created in the September of 2007 at Pauls Stradiņš Clinical University Hospital in Riga.

A noticeable increase of over 60% was noted in PAH patient incidence in Latvia in 2019 comparing to the reported incidence from 2018, which was 7.2 per MI and 9.0 per MAI.^6^ However, comparing the obtained results to a period of 2007–2016, where the PAH patient incidence in Latvia was 13.7 cases per MI, it is noted that overall, the PAH patient incidence has not increased, but fluctuates quite significantly year over year.^19^

The obtained data showed a predominance of NYHA functional class III or IV in PAH patients, where 20 patients (86.96%) had a NYHA functional class III or IV. Although notable advances have been made in the PAH treatment, the data shows that most of the patients have severe symptoms at the time of enrollment, indicating late PAH diagnosis. The NYHA functional class is a critical predictor of outcome in PAH, thus it indicates that the awareness of PAH in the medical field is still insufficient.^20^

Due to the varying incidence of different PH clinical groups, this study highlights the necessity for registries in each country or region of the world.^21^ Better screening and characterization of PH patients in well‐designed registries, which take into account local specificities, might enhance PAH detection and provide crucial information to health authorities.^21^

To summarize, the coordination between European PH registries and specialist centers will result in earlier PH diagnosis and improved access to a specific therapy.

## AUTHOR CONTRIBUTIONS

Ricards Kaulins takes responsibility for the content of the manuscript, including the data and analysis. Ainars Rudzitis, Aivars Lejnieks, Matiss Sablinskis, and Andris Skride were involved in study design, preparation of the draft manuscript, and critical revision and approval of the final manuscript.

## CONFLICT OF INTEREST

The authors declare no conflict of interest.

## ETHICS STATEMENT

This research was done in accordance with the 1975 Helsinki declaration (as revised in 2008). The study was approved by the Ethical Committee of the Pauls Stradiņš Clinical University Hospital (15.12.2009) Ethical Approval No. 151209‐6L.
